# A Rare Case of Pediatric Central Venous Catheter-Related Bloodstream Infection With Kocuria Varians

**DOI:** 10.7759/cureus.18200

**Published:** 2021-09-22

**Authors:** Aji Mathew, Jaidev R Nath, Ali Modaweb, Rubina Lone, Walid Abuhammour

**Affiliations:** 1 Pediatric Pulmonology, Al Jalila Children's Specialty Hospital, Dubai, ARE; 2 Pediatric Hospital Medicine, Al Jalila Children's Specialty Hospital, Dubai, ARE; 3 Pediatrics, Al Jalila Children's Specialty Hospital, Dubai, ARE; 4 Laboratory Medicine, Al Jalila Children's Specialty Hospital, Dubai, ARE; 5 Infectious Diseases, Al Jalila Children's Specialty Hospital, Dubai, ARE

**Keywords:** pediatric, kocuria, central line salvage, invasive bacterial infections, central line-associated infections (clabsi)

## Abstract

Cases of central line blood infections caused by *Kocuria *spp. are limited in the literature. Most of those infections have been detected in hospitalized patients with severe underlying disease or those with implanted catheters or with suppressed immunity. They are usually non-pathogenic in humans, but few cases of opportunistic infections in adult and pediatric populations have been reported. They can be serious in certain occasions. So treating physicians should not underestimate or ignore the significance of the infection with these bacteria.

We report a rare case of central venous catheter (CVC) infection associated with *Kocuria* *varians*, which was successfully treated with CVC salvage.

## Introduction

Central line-associated bloodstream infections (CLABSI) constitute a major part of hospital-acquired infections and may complicate treatment. Pathogens responsible for causing CLABSI include coagulase-negative *Staphylococcus *spp. (37.8%) followed by *Enterococcus *(11.2%), *Staphylococcus aureus* (9.3%), *Enterobacter *spp. (6.2%), *Candida albicans* (5.5%), *Pseudomonas aeruginosa* (4.9%), and *Klebsiella pneumonia *(4.1%) [1].

Cases of central line blood infections caused by *Kocuria *spp. are limited in the literature. Most of those infections have been detected in hospitalized patients with severe underlying disease or those with implanted catheters or with suppressed immunity.

*Kocuria *species are gram-positive cocci of the family *Micrococcaceae*. They are non-motile bacteria, which are catalase-positive and coagulase-negative [2]. They are widely distributed in the environment, and they are part of the normal skin and oral flora of humans. They are usually non-pathogenic in humans, but few cases of opportunistic infections in the adult and pediatric populations have been reported. It can be serious in certain occasions. A systematic review of the cases reported revealed that most of them are sensitive to certain antibiotics including Vancomycin [3]. So, treating physicians should not underestimate or ignore the significance of the infection with these bacteria.

We report a rare case of central venous catheter (CVC) infection associated with *Kocuria varians*, which was successfully treated and managed to salvage the CVC. However, most of the other cases reported ended up in the removal of the CVC due to the persistence of bacteria even after treatment.

## Case presentation

A six-month-old boy, a known case of short gut syndrome following bowel resection due to severe necrotizing enterocolitis (NEC) during neonatal period, was admitted with h/o fever for the one-day duration. He is a known case of syndrome 16 (Alpha thalassemia - intellectual disability syndrome), who had NEC during his neonatal period. He ended up in extensive bowel resection and subsequent short bowel syndrome. A Broviac line was placed three months prior, and he was on home total parenteral nutrition (TPN) with three to four feeds per day through gastrostomy. Fever was high grade and not associated with any other symptoms. In view of fever without any focus, the child was admitted and investigated. The total WBC count was 5.14 X 106, and neutrophils were 80%. CRP was 21 mg/L. Procalcitonin was 0.44 ng/mL. An electrolyte panel revealed mild hyponatremia with Na of 133 mmol/L. A respiratory viral PCR panel and COVID-19 PCR were negative. He was started empirically on Meropenem and Vancomycin as CVC infection was suspected. The urine routine was normal, and the urine culture was sterile.

Sample from both the central and peripheral lines flagged for growth in BactAlert (BioMérieux, Marcy-l'Étoile, France). Gram stain showed gram-positive cocci individually and in short chains. After inoculation onto appropriate media, the isolate was identified as *Kocuria varians* with 99% probability using the VITEK 2 gram-positive identification card (BioMérieux). Antibiotic susceptibility was done using Clinical and Laboratory Standards Institute (CLSI) M45 recommendation of media and antibiotic breakpoints. The organism was penicillin-resistant but sensitive to Vancomycin, Clindamycin, and Gentamicin. 

As bloodstream infections with *Kocuria* are rare and as there was no specific guideline for the treatment for *Kocuria*, we continued both Vancomycin and Meropenem. Repeat blood culture 48 hours after the initial one grew *Kocuria *from the CVC, but there was no growth from the peripheral blood. The child remained afebrile and asymptomatic, so the same antibiotics were continued. The echocardiogram did not reveal any vegetation or clots. An ultrasonogram of the abdomen did not reveal any collections. Subsequent blood cultures from both central catheter and peripheral blood were negative, so we stopped Meropenem and continued Vancomycin for a total of 14 days. The child remained afebrile throughout the hospital stay, and we were able to salvage the CVC.

## Discussion

*Kocuria *species are uncommon pathogenic organisms in humans. There are around 25 species of *Kocuria *identified to date [4]. Common species are *K. kristinae*, *K. varians*, *K. marina*, *K. rhizophila,* and *K. rosea*. They are catalase-positive and coagulase-negative gram-positive cocci seen in clusters. They are widely distributed in nature and have been found in normal skin and among oral cavity flora in humans and other animals [5]. Cases of *Kocuria *infections are limited in the literature. *Kocuria* species have been responsible for infections most commonly in immunocompromised hosts. *K. kristinae* was first described in 1974 and has been reported to cause catheter-related bacteremia [6]. Most *Kocuria *species, except for *K. kristinae*, are strict aerobes. Even though *Kocuria *species were considered as non-pathogenic bacteria in humans initially, there is an increasing incidence of superficial and deep-seated invasive infections recently. There are reports of infective endocarditis, osteomyelitis, CLABSI, brain abscess, peritonitis, and meningitis caused by *Kocuria* species [7]. There is a recent case report of infective endocarditis caused by *K. rosea* in a 10-year-old immunocompetent female [8]. Pierron et al, has recently reported a case of catheter-related bacteremia and endocarditis with *K. rhizophila* in an 81-year-old diabetic patient [9].

In the pediatric population, *K. kristinae* and *K. varians* have caused central line infections in premature babies, immunocompetent patients, and immunocompromised patients with long-term intravenous catheters [10]. Brain abscess caused by *K. varians* has also been reported [11].

Purty et al. in 2013 has reviewed almost all reported cases of significant *Kocuria* infections. In their study, most infections were confirmed to be due to the species *K. kristinae*, and 11 cases out of the total (n = 20) were bloodstream infections. The remaining cases recognized other *Kocuria *species as etiologic agents: *K. varians* (n = 3), *K. rosea* (n = 2), *K. rhizophila *(n = 2), and *K. marina *(n = 2) [12]. The majority of cases of *Kocuria*-related peritonitis occurred in patients undergoing continuous ambulatory peritoneal dialysis (CAPD) [13].

There are no specific treatment guidelines for the management of *Kocuria *as there are limited numbers of cases reported. Pathogenic *Kocuria *are highly susceptible to broad-spectrum antibiotics. Most of them are resistant to ampicillin and erythromycin [14]. Susceptibility was highest to Vancomycin, Linezolid, Teicoplanin, Cefotaxime, and Meropenem [2]. Monotherapy with Vancomycin, Oxacillin, and Piperacillin-Tazobactam or combination therapy with Teicoplanin and Vancomycin, Vancomycin and Meropenem, and Ciprofloxacin and Clindamycin has been used successfully before.

In our case, blood was inoculated in blood agar plate (BAP), MacConkey agar (MAC), and chocolate agar (CA). After 48 hours, there were yellowish colonies that were catalase-positive and Staph latex-negative. The isolate was identified as *K. varians* with 99% probability using the VITEK 2 gram-positive identification card. The sample was also sent to a referral laboratory that confirmed *K. varians* using matrix-assisted laser desorption and ionization time of flight mass spectrometry.

In the setting of a CVC infection, our practice is to proceed with CVC removal for any of the following situations [15]:

1. A positive blood culture for *S. aureus* or any fungal species

2. Persistent fever or positive blood cultures with any organism after 72 hours of appropriate antibiotic therapy

3. Development of serious complications (e.g., suppurative thrombophlebitis, endocarditis or other metastatic infection, septic shock, etc.)

Most of the cases of central venous catheter-associated infections by *Kocuria* species reported ultimately led to the removal of the central line due to the persistence of positive blood cultures. Bhavsar et al. reported a seven-month-old child with short gut syndrome with *K. varians* bacteremia associated with CVC. The persistence of infection even after treatment with Vancomycin led to the removal of the Broviac catheter [10]. Even though they expressed Vancomycin sensitivity in vitro, drug administration did not change the clinical picture and failed to eradicate the bacteria in most of the cases, most likely due to the formation of biofilm. 

In our case, the first and second cultures showed growth of *K. varians*, but the subsequent blood cultures were negative. With a course of Vancomycin for 14 days, we succeeded in clearing CVC-associated bacteremia and were able to salvage the central line. To the best of our knowledge, our patient represents a rare case of catheter-related bacteremia by *K. varians* and the first successful case of catheter salvage.

## Conclusions

*Kocuria *still remains a rare bacterium that causes significant infections in humans, but there is an increasing incidence of serious infections with them in the immunocompromised host. CVC-related bacteremia, endocarditis, and peritonitis remain the most common infections by *Kocuria*. Clinicians should not underestimate the importance of *Kocuria*, especially in cases where the patient has an implanted medical device or catheters.
